# Lung ultrasound to evaluate aeration changes in response to recruitment maneuver and prone positioning in intubated patients with COVID-19 pneumonia: preliminary study

**DOI:** 10.1186/s13089-023-00306-9

**Published:** 2023-01-25

**Authors:** Gianmaria Cammarota, Andrea Bruni, Giulio Morettini, Leonardo Vitali, Francesco Brunelli, Filippo Tinarelli, Rachele Simonte, Elisa Rossi, Matteo Bellucci, Giacomo De Girolamo, Antonio Galzerano, Luigi Vetrugno, Salvatore M. Maggiore, Elena Bignami, Danila Azzolina, Olivia Dow, Paolo Navalesi, Edoardo De Robertis

**Affiliations:** 1 Department of Medicine and Surgery, Università degli Studi di Perugia, Perugia, Italy; 2 Anestesia and Intensive Care Service 2, Azienda Ospedaliera di Perugia, Perugia, Italy; 3 Anesthesia and Intensive Care, Department of Medical and Surgical Sciences, Magna Græcia University, Catanzaro, Italy; 4 Department of Anesthesiology and Intensive Care, Ospedale SS Annunziata & Department of Innovative Technologies in Medicine e Odontostomatology, Università Gabriele D’Annunzio di Chieti-Pescara, Chieti, Italy; 5 Anesthesiology, Critical Care and Pain Medicine Division, Department of Medicine and Surgery, University of Parma, Parma, Italy; 6 Department of Medical Science, University of Ferrara, Ferrara, Italy; 7 Surrey and Sussex NHS Healthcare Trust, Redhill, UK; 8 Department of Medicine, University of Padova, Padua, Italy

**Keywords:** Acute respiratory distress syndrome, COVID-19, Lung ultrasound

## Abstract

**Background:**

This single-center preliminary prospective observational study used bedside ultrasound to assess the lung aeration modifications induced by recruitment maneuver and pronation in intubated patients with acute respiratory disease syndrome (ARDS) related to coronavirus 2019 disease (COVID-19).

All adult intubated COVID-19 patients suitable for pronation were screened. After enrollment, patients underwent 1 h in a volume-controlled mode in supine position (baseline) followed by a 35-cmH_2_O-recruitment maneuver of 2 min (recruitment). Final step involved volume-controlled mode in prone position set as at baseline (pronation). At the end of the first two steps and 1 h after pronation, a lung ultrasound was performed, and global and regional lung ultrasound score (LUS) were analyzed. Data sets are presented as a median and 25th–75th percentile.

**Results:**

From January to May 2022, 20 patients were included and analyzed. Global LUS reduced from 26.5 (23.5–30.0) at baseline to 21.5 (18.0–23.3) and 23.0 (21.0–26.3) at recruitment (*p* < 0.001) and pronation (*p* = 0.004). In the anterior lung regions, the regional LUS were 1.8 (1.1–2.0) following recruitment and 2.0 (1.6–2.2) in the supine (*p* = 0.008) and 2.0 (1.8–2.3) in prone position (*p* = 0.023). Regional LUS diminished from 2.3 (2.0–2.5) in supine to 2.0 (1.8–2.0) with recruitment in the lateral lung zones (*p* = 0.036). Finally, in the posterior lung units, regional LUS improved from 2.5 (2.3–2.8) in supine to 2.3 (1.8–2.5) through recruitment (*p* = 0.003) and 1.8 (1.3–2.2) with pronation (*p* < 0.0001).

**Conclusions:**

In our investigation, recruitment maneuver and prone positioning demonstrated an enhancement in lung aeration when compared to supine position, as assessed by bedside lung ultrasound.

*Trial registration*: www.clinicaltrials.gov, Number NCT05209477, prospectively registered and released on 01/26/2022.

## Introduction

In coronavirus 2019 disease (COVID-19) patients undergoing invasive mechanical ventilation (IMV), prone positioning has been adopted as a rescue therapy to improve oxygenation [1]. In conventional acute respiratory distress syndrome (ARDS) switching from supine to prone position allows the achievement of a more homogenous gas-to-tissue ratio distribution across the lung by releasing the dorsal atelectasis at expense of the ventral zones [2]. However, lung collapse redistribution is a phenomenon mainly observed in early ARDS [3, 4]. As recently described in COVID-19 ARDS, the extent of atelectasis redistribution is strongly related to the amount of consolidated tissue present in the dorsal lung regions [5]. Thus, the response to the prone position and recruitment maneuver relies on the extent of consolidation present in the posterior lungs, which is increased in the advanced stages of the disease [5, 6]. In intubated COVID-19 ARDS patients, the assessment of the lung reaeration secondary to prone position and recruitment maneuver has been commonly evaluated through computer-tomography (CT) scans [5, 7]. In COVID-19 ARDS, lung ultrasound has been recommended as a lung monitoring tool during IMV [8]. In conventional and COVID-19 ARDS, the lung ultrasound score (LUS) is a reliable tool for the assessment of global and regional lung aeration [9–13]. Accordingly, we hypothesized that lung ultrasound could be employed in the evaluation of lung aeration following recruitment maneuver and prone position in intubated patients suffering from ARDS related to COVID-19.

The primary aim of the present single-center preliminary investigation was the evaluation of lung aeration in response to recruitment maneuver and prone positioning, through the use of bedside lung ultrasound.

## Methods

The present analysis, registered at www.clinicaltrials.com (NCT05209477, released on 01/26/2022), was conducted on prospectively collected data describing the clinical course of COVID-19 patients admitted to the ICU of Perugia University Hospital, Italy, following the approval by the local ethical committee (Protocol No. 3658/20). The study was performed in line with the Helsinki Declaration principles. Written informed consent was waived due to the observational nature of the study. All patients were treated according to the standard clinical practice and local institutional protocol.

### Enrollment

From January to May 2022, all critically ill adult patients undergoing IMV with sedation and muscular paralysis for ARDS related to COVID-19 and suitable for prone positioning as a rescue therapy were screened. Concurring with the local institutional protocol, the decision was made to prone patients when the partial arterial oxygen tension on inspired oxygen fraction ratio (PaO_2_/FiO_2_) was < 150 mmHg following intubation by an attending physician [14]. Exclusion criteria included: pregnancy, inability to obtain a complete lung ultrasound assessment due to difficult sonographic windows, any contraindication to prone position [15], pneumothorax and pneumomediastinum, chronic obstructive pulmonary disease, any contraindication to recruitment maneuver [16], hemodynamic instability [17], prone position application after 3 days from IMV onset [7].

### Study protocol

Enrolled patients were initially ventilated using a volume-controlled setting in the supine position (baseline) to achieve tidal volumes of 6 to 8 ml/kg [5] of predicted body weight. In addition, positive end-expiratory pressure (PEEP) was applied in combination with an inspired oxygen fraction (FiO_2_) defined by low PEEP–FiO_2_ tables to achieve peripheral oxygen saturations (SpO_2_) of 88–95% [18].

After the onset of 1 h of protective ventilation, a 2-min-lasting recruitment maneuver was administrated in the pressure-controlled ventilation mode (recruitment) with a total inspiratory pressure of 35 cmH_2_O [5]. The PEEP and FiO_2_ remained as set up during baseline step and the mechanical respiratory rate was set to 10 breaths/min with an inspiration-to-expiration ratio of 1:1. Subsequently, patients were proned whilst remaining on the same ventilator settings as in baseline step (pronation).

### Measurements

Before the study enrollment, the following demographic and clinical data were collected: age, gender, predicted body weight, PaO_2_/FiO_2_ after intubation, comorbidities, days spent with NIRS before intubation, infection diagnosis to intubation delay, hospital admission to intubation delay, IMV duration, sequential organ failure assessment (SOFA) score, PEEP, and FiO_2_. Following completion of STEP1, 2, and 1 h of prone position lung ultrasound and arterial blood gases (ABGs) analysis were carried out whilst also assessing respiratory system mechanics and hemodynamic status. ABGs analysis was performed to assess pH, PaO_2_, PaO_2_/FiO_2_, and partial arterial carbon dioxide tension (PaCO_2_).

Expiratory tidal volume and respiratory rate values were obtained from the ventilator [19] at the end of each step and respiratory system compliance along with driving pressure were computed. Vital signs were continuously assessed for the whole study duration, monitoring the SpO_2_, invasive arterial blood pressure, heart rate, and ECG.

### Technical components

Lung ultrasound was performed at the bedside as previously described [20–22], using a portable ultrasound machine equipped with both 2.0–4.0 MHz-convex and 7.5–12.0 MHz-linear probes (MylabX6, Esaote SPA, Italy). Six quadrants for each hemithorax were scanned: the superior and inferior parts of the anterior, lateral, and posterior regions of the chest wall. In each region, LUS and the corresponding aeration pattern were computed as previously indicated [20–22]: A-line alone or in combination with less than 3 B lines (0 point—normal aeration pattern); B lines present in less than 50% of the pleural line (1 point—B1 aeration pattern); B lines present in more than 50% of the pleural line (2 points—B2 aeration pattern); total loss of aeration suggestive for lung consolidation (3 points—C aeration pattern). Accordingly, global and regional LUS were computed. The global LUS was defined as the sum of the scores obtained in the 12 sonographic lung regions and varied from a minimum of 0 (normal aeration pattern) to a maximum of 36 (complete loss of aeration) [20–22]. The regional LUS was computed for the anterior, lateral, and posterior regions of interest as well as the superior and inferior regions [20]. The regional LUS corresponded to the mean score of all pertaining intercostal spaces of each region and ranged from a minimum of 0 points to a maximum of 3 points.

The ultrasonography assessors were not involved in patients’ care. In addition, ultrasonographic and clinical data were independently gathered and stored by a data collector, not involved in the ultrasound assessment and patients’ care.

### Statistical analysis

According to previous findings [19], to observe a reduction of LUS from 22 ± 3 in supine position to 20 ± 4.9 in prone position, a total sample size of 20 subjects was computed (Type I error rate of 0.05 and a Type II error rate of 0.20, 80% power).

Continuous variables were described as median and 25th–75th interquartile range. The comparison between all the study steps was performed by Friedman’s test for nonparametric repeated measures and Post Hoc test with Bonferroni’s correction. To assess the effects of ventilatory strategy (supine, recruitment, prone) and lung region of interest (anterior, lateral, and posterior–superior and inferior) on the dependent variable, a generalized mixed model analysis with Satterthwaite methods for degrees of freedom and Post Hoc test with Bonferroni’s correction were employed. A generalized linear mixed model (GLMM) was estimated on the observed data. The graphical representation of the GLMM predicted values of PaO_2_/FiO_2_ according to LUS has been reported together with the 95% confidence bounds. Two-tailed tests were applied for hypothesis testing and statistical significance was considered for *p* values < 0.05. Statistical analyses were carried out through R3.5.2 software (The R Foundation).

## Results

**Fig. 1:**
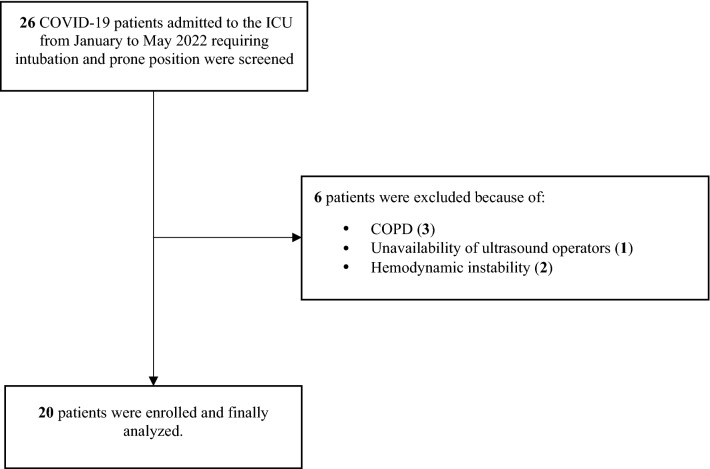
Enrollment flow diagram. COVID-19, disease related to coronavirus 2019; COPD, chronic obstructive pulmonary disease

From January to May 2022, 26 critically ill adult COVID-19 patients undergoing IMV and prone positioning were screened of whom 20 were enrolled and analyzed (Fig. 1). The baseline clinical characteristics of the study population are reported in Table 1. Three patients received 1 pronation attempt before the study day.

**Table 1:** Baseline clinical characteristics of the study population

Variable	Study population
N	20
Male (%)	95
Age (years)	69.0 (63.0–74.3)
Body mass index (kg/m^2^)	27.2 (26.0–33.2)
Predicted body weight (kg)	70.6 (69.5–74.3)
Infection diagnosis to intubation delay (days)	10.0 (9.0–13.0)
Hospital admission to intubation delay (days)	4.0 (2.0–7.0)
NIRS pre-intubation (days)	2.5 (1.8–5.3)
IMV duration pre-enrollment (days)	1.0 (0.0–2.0)
Sequential organ failure assessment score	5.5 (4.0–8.5)
PaO_2_/FiO_2_ post-intubation (mmHg)	77.0 (67.8–98.0)
PEEP at enrollment	10.0 (10.0–12.0)
FiO_2_ at enrollment	0.7 (0.7–0.8)
Pronation attempts (N)	0.0 (0.0–0.0)
Comorbidities	
Arterial hypertension (%)	85
Chronic heart disease (%)	20
Diabetes (%)	30
Chronic kidney failure (%)	10

Data are presented as percentage or median and 25–75th percentile.

*NIRS* non-invasive respiratory support, *IMV* invasive mechanical ventilation, *PaO*_*2*_*/FiO*_*2*_ partial arterial oxygen tension on inspired oxygen fraction ratio, *PEEP* positive end-expiratory pressure

Respiratory mechanics, ABGs, and hemodynamics are presented in Table 2. As expected, the application of a recruitment maneuver increased the driving pressure, plateau pressure, and tidal volume with respect to supine and prone position (*p* < 0.001 for all comparisons), whereas no modifications were observed in the respiratory system compliance. Mechanical respiratory rate diminished with recruitment maneuver compared to the supine and prone position (*p* < 0.001 for all comparisons), as per study protocol. PaO_2_/FiO_2_ values progressively improved switching from supine to recruitment (*p* = 0.022) and from recruitment to prone position (*p* = 0.008), where PaO_2_/FiO_2_ was higher compared to supine (*p* < 0.001), respectively. PaCO_2_ and pH reduced with recruitment maneuver and prone positioning compared to supine (PaCO_2_: *p* < 0.001 and *p* = 0.010; pH: *p* < 0.001 and *p* = 0.013). Hemodynamics did not change across all the study steps.

**Table 2:** Respiratory mechanics, arterial blood gases, and hemodynamics

Parameters	Supine (*n*.20)	Recruitment (*n*.20)	Prone (*n*.20)	*P* value
Respiratory mechanics				
Respiratory system compliance (ml/cmH_2_O)	38.5 (28.5–48.0)	32.0 (27.0–45.0)	37.0 (31.0–48.5)	0.377
Respiratory system driving pressure (cmH_2_O)	13.0 (10.0–16.0)	25.0 (23.0–25.0)^a^	13.5 (9.8–16.0)^b^	< 0.001
Plateau pressure (cmH_2_O)	24.0 (20.0–27.0)	35.0 (35.0–35.0)^a^	24.0 (21.0–26.3)^b^	< 0.001
Tidal volume (ml/kg)	6.7 (6.4–7.0)	11.2 (9.5–14.9)^a^	6.6 (6.1–6.9)^b^	< 0.001
Mechanical respiratory rate (breaths/min)	22.0 (20.0–24.3)	10.0 (10.0–10.0)^a^	21.0 (20.0–24.3)^b^	< 0.001
Arterial blood gases				
PaO_2_/FiO_2_ (mmHg)	111.0 (80.3–139.0)	114.0 (95.5–189.0)^c^	156.0 (139.0–204.0)^a^,^d^	< 0.001
PaCO_2_ (mmHg)	57.0 (44.0–68.3)	51.0 (43.5–56.0)^a^	50.5 (42.8–61.0)^e^	< 0.001
pH	7.36 (7.28–7.39)	7.39 (7.34–7.44)^a^	7.37 (7.31–7.43)^f^	< 0.001
Hemodynamics				
Mean arterial pressure (mmHg)	88.0 (74.0–93.3)	80.0 (72.5–87.0)	82.5 (79.8–88.5)	0.264
Heart rate (beats/min)	81.5 (60.0–101.0)	77.5 (60.8–104.0)	81.5 (68.5–101.0)	0.499

Data are presented as median and 25th–75th percentile

*PaO*_*2*_*/FiO*_*2*_ partial arterial oxygen tension on inspired oxygen fraction ratio, *PaCO*_*2*_ partial arterial carbon dioxide tension

^a^vs supine, *p* < 0.001

^b^vs recruitment, *p* < 0.001

^c^vs supine, *p* = 0.022

^d^vs recruitment, *p* = 0.008

^e^vs supine, *p* = 0.010

^f^vs supine, *p* = 0.013

**Fig. 2:**
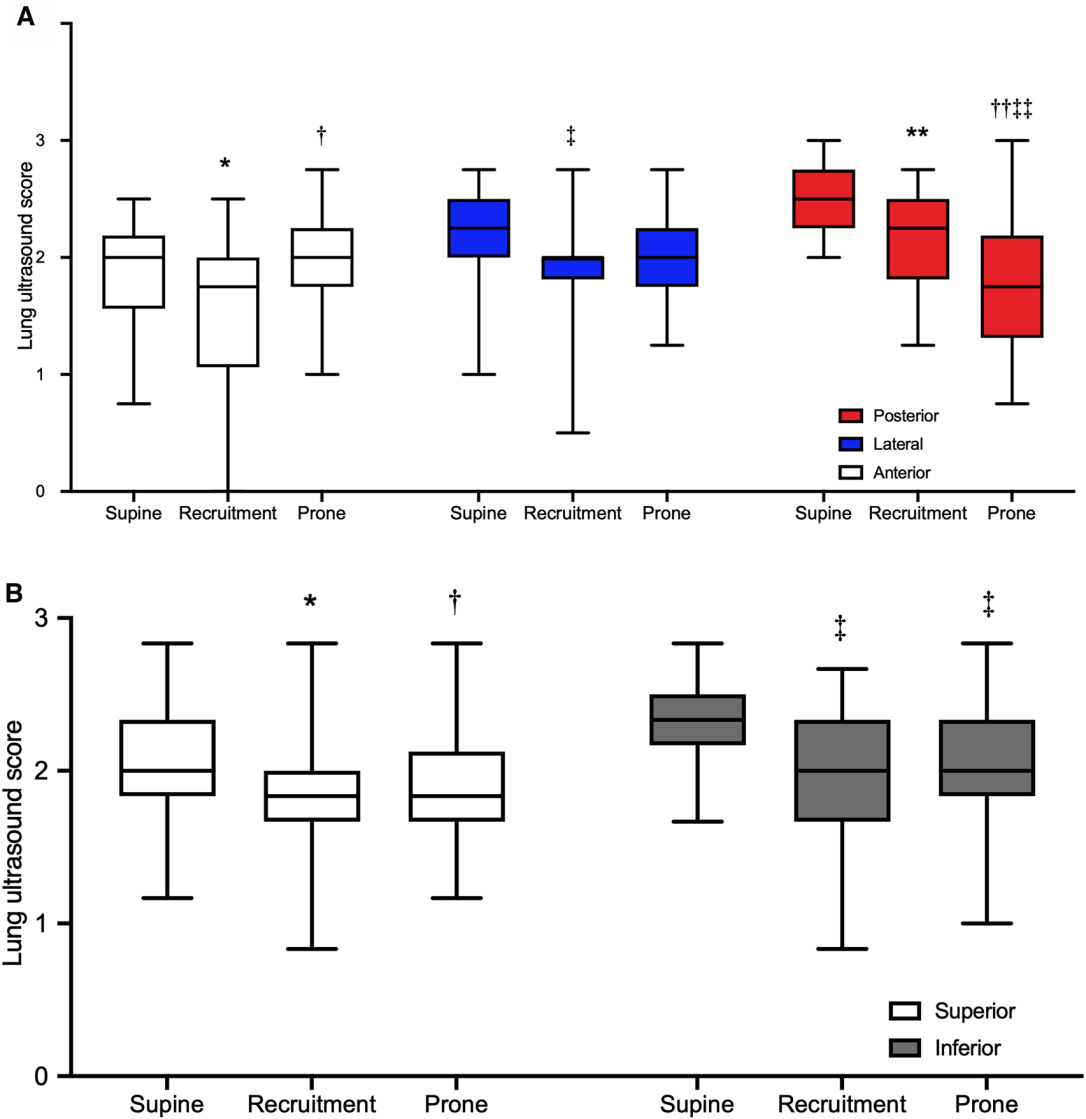
Regional lung ultrasound score. **A** Regional ultrasound score for anterior, lateral, and posterior regions of interest regardless of body side. Data are present as boxes (median and 25th–75th percentile) and whiskers (minimum to maximum) for anterior, lateral, and posterior regions of interest at supine, recruitment, and prone position. * vs supine, *p* = 0.008; † vs recruitment, *p* 0.023; ‡ vs supine. *p* = 0.036; ** vs supine, *p* = 0.003; †† vs supine, *p* < 0.0001; ‡‡ vs recruitment, *p* = 0.002. **B** Regional ultrasound score for superior and inferior regions of interest regardless of the body side. Data are present as boxes (median and 25th–75th percentile) and whiskers (minimum to maximum) for superior and inferior regions of interest at supine, recruitment, and prone position. * vs supine, *p* < 0.001; † vs supine, *p* 0.024; ‡ vs supine, *p* < 0.0001

**Fig. 3:**
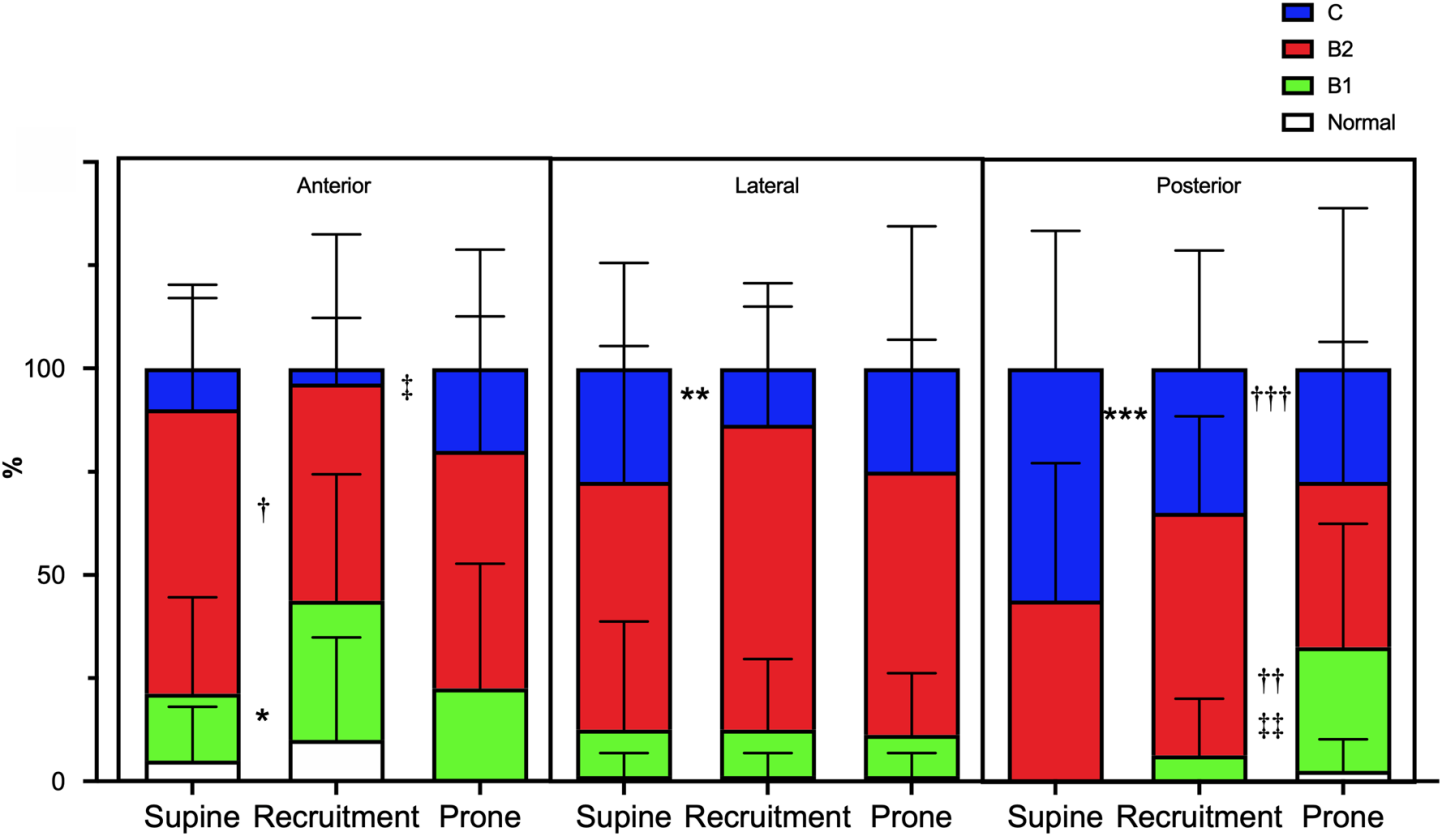
Regional aeration pattern. Regional aeration pattern for anterior, lateral, and posterior regions of interest regardless of the body side. Aeration distribution considering all the lung ultrasound patterns (0–3) lung are expressed as mean and standard deviation for each region of interest at supine, recruitment, and prone position, regardless of body side. Normal aeration pattern (lung ultrasound score 0—white); B1 aeration pattern (lung ultrasound score 1—light grey); B2 aeration pattern (lung ultrasound score 2—dark grey); C aeration pattern (lung ultrasound score 3—ultra-dark grey). Anterior region of interest: * vs supine for B1, *p* = 0.039; † vs supine for B2, p 0.022; ‡ vs recruitment for C, *p* = 0.030. Lateral region of interest: ** vs supine for C, *p* = 0.020. Posterior region of interest: †† vs supine for B1, *p* < 0.001; ‡‡ vs recruitment for B1, *p* = 0.004; *** vs supine for C, *p* = 0.016; ††† vs supine for C, *p* = 0.033

Table 3 describes LUS. Global LUS diminished with recruitment maneuver and prone position with respect to supine (*p* < 0.001 and *p* = 0.004). Moreover, recruitment maneuver caused a greater improvement in global LUS compared to prone position (*p* = 0.002). In the generalized mixed model analysis, LUS was not dissimilar when the interaction between intervention (supine, recruitment, prone), lung region (anterior, lateral, posterior–superior, inferior), and body side (left, right) was considered. Figure 2 depicts the regional LUS according to the generalized mixed model analysis based on interaction amongst the intervention and lung regions of interest regardless of the body side. As depicted in Fig. 2 A, recruitment maneuver reduced regional LUS with respect to supine (*p* = 0.008) and prone position (*p* = 0.023) in anterior lung regions, as well as to supine, in lateral lung regions (*p* = 0.036). In the posterior regions, regional LUS progressively decreased switching from supine to recruitment maneuver (*p* = 0.003) and from recruitment to prone position (*p* < 0.0001), where regional LUS was lower than in the supine position (*p* = 0.002). Moreover, regional LUS was higher for the supine compared to the recruitment maneuver and prone for both superior (vs recruitment *p* < 0.001; vs prone *p* = 0.024) and inferior lung regions (*p* < 0.0001 for all comparisons). The aeration pattern distribution across all study steps is represented in Fig. 3. In the anterior lung regions, recruitment maneuver induced an increase in B1 pattern as well as a reduction in B2 pattern when compared to supine (*p* = 0.039 and *p* = 0.022). The same lung regions showed a worsening C pattern moving from recruitment to prone position (*p* = 0.030). In the lateral lung regions, recruitment improved the C pattern with respect to supine (*p* = 0.020). Finally, in the posterior lung regions, the B1 pattern was more pronounced in the prone position compared to supine (*p* < 0.001) and recruitment (*p* = 0.004), whereas the C pattern diminished with recruitment and prone position with respect to supine (*p* = 0.016 and *p* = 0.033).

**Table 3:** Lung ultrasound

Parameters	Supine (*n*.20)	Recruitment (*n*.20)	Prone (*n*.20)	*P* value
Global lung ultrasound score	26.5 (23.5–30.0)	21.5 (18.0–23.3)^a^	23.0 (21.0–26.3)^bc^	< 0.001
Lung ultrasound score				0.726
Right lung				
Antero-superior region	2.0 (1.0–2.0)	2.0 (1.0–2.0)	2.0 (2.0–2.0)	
Antero-inferior region	2.0 (2.0–2.0)	2.0 (1.0–2.0)	2.0 (2.0–3.0)	
Latero-superior region	2.0 (2.0–2.0)	2.0 (1.0–2.0)	2.0 (2.0–2.0)	
Latero-inferior region	2.0 (2.0–2.3)	2.0 (2.0–2.3)	2.0 (2.0–2.0)	
Postero-superior region	2.0 (2.0–3.0)	2.0 (2.0–2.0)	2.0 (1.0–2.0)	
Postero-inferior region	3.0 (2.8–3.0)	2.0 (2.0–3.0)	2.0 (1.0–2.0)	
Left lung				
Antero-superior region	2.0 (1.5–2.0)	1.0 (1.0–2.0)	2.0 (1.0–2.0)	
Antero-inferior region	2.0 (2.0–2.0)	2.0 (1.5–2.0)	2.0 (2.0–2.0)	
Latero-superior region	2.0 (2.0–2.0)	2.0 (2.0–2.0)	2.0 (2.0–2.0)	
Latero-inferior region	2.0 (2.0–3.0)	2.0 (2.0–2.0)	2.0 (2.0–2.0)	
Postero-superior region	2.0 (2.0–3.0)	2.0 (2.0–3.0)	2.0 (1.0–2.0)	
Postero-inferior region	3.0 (2.0–3.0)	2.0 (2.0–3.0)	2.0 (1.0–2.0)	

Data are presented as median an 25th–75th percentile

^a^vs supine, *p* < 0.001

^b^vs recruitment, *p* = 0.002

^c^vs supine, *p* = 0.004

**Fig. 4:**
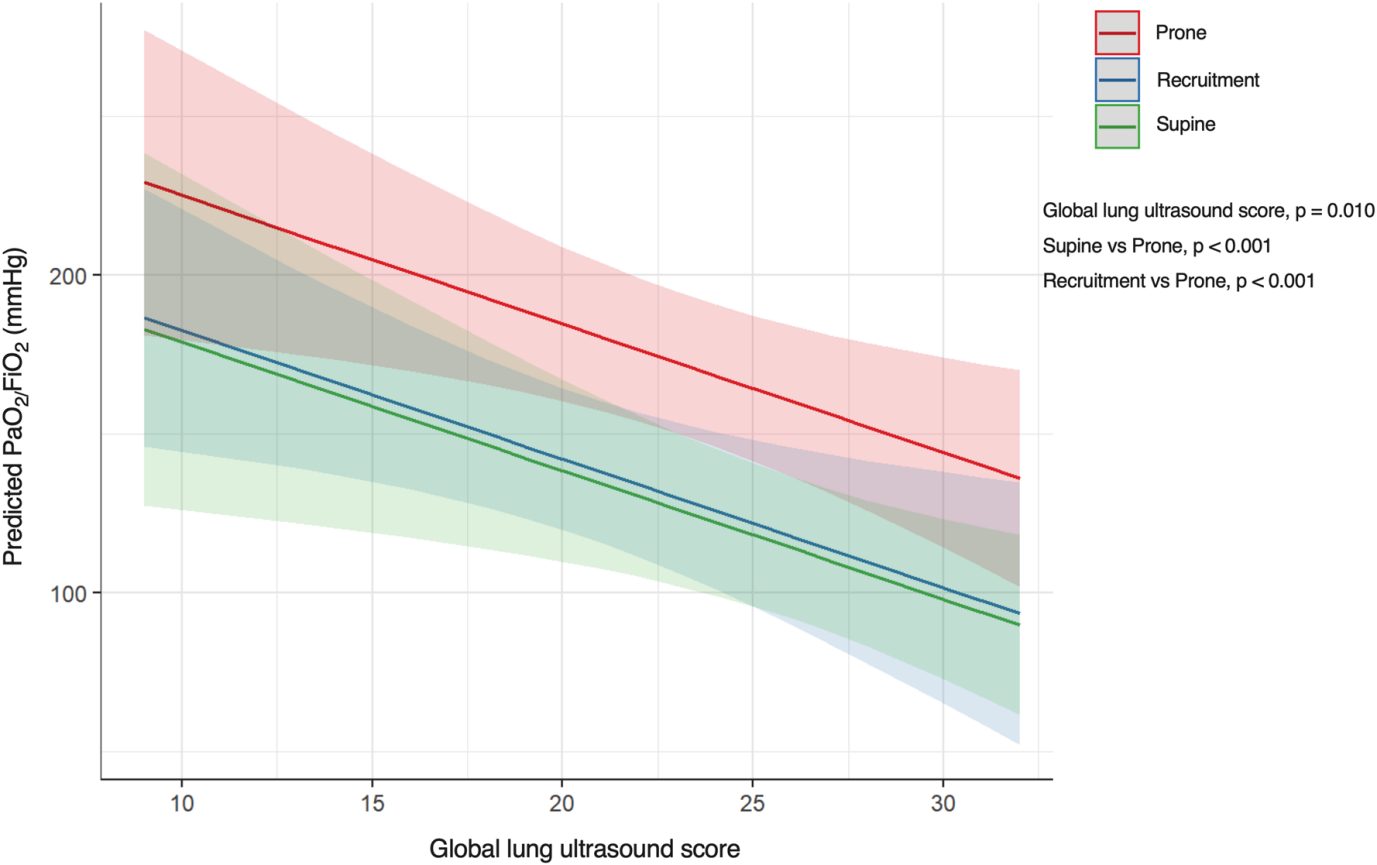
Predicted PaO_2_/FiO_2_ at varying global lung ultrasound scores in response to supine, recruitment maneuver, and prone position. Predicted PaO_2_/FiO_2_ modifications according to global lung ultrasound score with 95% confidence intervals adjusted for interventions, i.e., prone position (red), recruitment (blue), and supine (green) are depicted. Fixed effect global lung ultrasound score estimate (95% CI) = − 4.1 (− 7.0 to − 1.1); *p* = 0.010. Fixed effect prone vs supine estimate (95% CI) = 46.17 (26.8 to 65.6); *p* < 0.001. Fixed effect prone vs recruitment estimate (95% CI) = 42.5 (26.8 to 65.6); *p* < 0.001. PaO_2_/FiO_2_, partial arterial oxygen tension on inspired oxygen fraction ratio

The trends of predicted PaO_2_/FiO_2_ at varying global LUS in response to supine, recruitment, and prone position are displayed in Fig. 4. Overall, predicted PaO_2_/FiO_2_ values reduced with the rise of global LUS regardless of the study conditions (*p* = 0.010). However, predicted PaO_2_/FiO_2_ was higher in the prone position compared to supine and recruitment (*p* < 0.001 for all comparisons).

## Discussion

The main findings of the present single-center preliminary investigation can be summarized as follows: (1) overall, recruitment maneuver and prone position improved global and regional LUS; (2) Recruitment maneuver led to improved regional lung aeration patterns in most of the sonographic regions of interest, by increasing B1 pattern in anterior regions and reducing C pattern in lateral and posterior regions; (3) Despite worsening in lung aeration in anterior lung regions, the prone position enhanced regional lung aeration pattern in the posterior lung units by increasing B1 pattern and diminishing C pattern.

The variability of the response to recruitment and prone position is high in COVID-19 patients, despite the same degree of hypoxemia [5, 7, 23]. As recently reported [5], in the early stages of COVID-19 ARDS, a 35-cmH_2_O-recruitment maneuver was usefully employed to reduce the atelectatic lung tissue distribution compared to 5-cmH_2_O-ventilation in the supine position. In addition, a more homogeneous gas-to-tissue ratio was achieved by prone positioning compared to supine owing to the re-expansion of the dorsal lung units at the expense of ventral atelectasis [5, 7]. This response can be altered depending on the superimposed pressure gradient across the lung [24], the shape of the lung and the chest wall, the compression of the lung by the abdomen and heart, the compliance of the non-dependent and dependent chest wall, and the vertical distribution of the lung mass.

In keeping with previous findings [5], however, the effects exerted on atelectasis by recruitment maneuver and prone position are strongly related to lung disease history. Undeniably, significant lung consolidation and fibrotic changes are observed in the advanced stages of COVD-19 ARDS, reducing the recruitability of lung tissue through maneuvers, when compared to early phases of the disease.

In our COVID-19 patients’ cohort, we observed that recruitment maneuver exerted its effects by improving aeration in the anterior, lateral, and posterior regions of the lungs. In turn, the prone position enhanced the posterior lung aeration at the expense of the anterior lung regions, where atelectasis was increased, probably as a consequence of increased superimposed pressure as previously described [5, 7].

The response in oxygenation to recruitment maneuver and prone position is attributable to the balance of lung recruitment/de-recruitment and the modifications of lung perfusion. In particular, the variations in oxygenation following a 35-cmH_2_O recruitment maneuver will be reliant on the balance between the perfusion of the re-expanded lung units and the degree of diverted blood flow to the consolidated lung zones [5]. In the prone position, the gravitational blood flow diversion to the ventral atelectatic regions counterbalances the oxygenations alterations induced by alveolar recruitment [5]. Furthermore, the pulmonary perfusion distribution is variously affected by COVID-19 [25, 26].

In our series, recruitment maneuver and prone position improved overall oxygenation as described by PaO_2_/FiO_2_ modifications observed. In addition, contrary to previous findings [5, 7], we observed a reduction of PaCO_2_ with recruitment maneuver and prone position compared to supine. During the interpretation of our results, it is worth considering the history of the disease with the consequent implications on the lung recruitability, and NIRS duration before intubation. Undoubtedly, our population was studied at an earlier stage of the disease and with fewer days spent on NIRS than elsewhere described [5]. Thus, our cohort of COVID-19 patients might have experienced less patients-self-induced lung injury. In addition, the median PEEP of 10 cmH_2_O set in our study according to low PEEP–FiO_2_ tables was different with respect to previous investigations [5, 7]. Thus, it is presumed that our approach was more considerate of the disease and lung recruitability, as previously observed in conventional ARDS [27], compared to a fixed 5-cmH_2_O-PEEP strategy [5] or a PEEP chosen at the discretion of the attending physician [7].

The strength of the present paper consists in highlighting the usefulness of lung ultrasound to assess lung aeration modifications in response to recruitment maneuver and prone position, at the patient’s bedside in early ARDS related to COVID-19. In the context of a pandemic, where work overload and infection control restrictions may not allow for the easy attainment of advanced radiological investigations, such as computer-tomography scans; this is extremely relevant.

The present investigation has several limitations as discussed in the following paragraph. This study was a single-center investigation. Although the computed sample size was based on PaO_2_/FiO_2_ modifications switching from supine to prone position, it was suitable to describe the lung ultrasound changes across all study steps. In interpreting our data, it is worth to consider the difference between conventional ARDS and COVID-19-related ARDS in terms of uncoupling between clinical presentation and anatomical characteristics of the lung due to the involvement of lung perfusion mainly at an early stage [28]. The cohort population of this study was not standardized for the COVID-19 ARDS phenotype or disease history. In addition, patients of this cohort study may have undergone one to two pronation attempts before the study enrollment. As a consequence, the response to maneuvers performed during the study might be affected by previous pronation attempts. We employed quantitative lung ultrasound to assess the lung aeration in our patients’ population. This tool has demonstrated a good diagnostic accuracy for COVID-19 pneumonia when compared to CT scan [13]. However, LUS has been introduced in pre-COVID-19 era for quantification of lung aeration. Thus, the irregular distribution of the interstitial involvement and consolidation alternating with spared areas at lung sonographic examination may raise several concerns on the accuracy of the LUS in assessing pulmonary aeration in COVID-19-related ARDS [29]. In addition, a new LUS examination relying on the evaluation of the pulmonary lesion extension and not the degree of lung aeration may be usefully employed to follow the progression of COVID-19 disease and personalize the treatments [29, 30].

In our series, we did not evaluate lung aeration and perfusion with computer-tomography scans or advanced respiratory monitoring tools, such as using electrical impedance tomography. Accordingly, we were not able to provide data and draw any conclusion about global and regional lung overdistension as well as lung perfusion modifications occurring at any time during the study. Due to it being a single-center analysis, further multicenter trials are required.

## Conclusions

In our single-center preliminary observational study, as assessed through bedside lung ultrasound, recruitment maneuver improved lung aeration in the most of lung regions evaluated, whereas prone position enhanced the posterior lung regions’ aeration at the expense of the anterior lung regions.

## Data Availability

The data of the present investigation are available, upon reasonable request, by contacting the corresponding author.

## Abbreviations

ABGs: Arterial blood gases
ARDS: Acute respiratory distress syndrome
COVID-19: Coronavirus 2019 disease
FiO2: Inspiratory oxygen fraction
IMV: Invasive mechanical ventilation
LUS: Lung ultrasound score
NIRS: Non-invasive respiratory support
PaO2: Arterial oxygen tension
PaO_2_/FiO_2_: Partial arterial oxygen tension on inspired oxygen fraction
PaCO_2_: Partial arterial carbon dioxide tension
PEEP: Positive end-expiratory pressure
SOFA: Sequential organ failure assessment
SpO_2_: Peripheral oxygen saturation
